# Nonlinear Optical and Ion Sensor Properties of Novel Molecules Conjugated by Click Chemistry

**DOI:** 10.3390/polym14081516

**Published:** 2022-04-08

**Authors:** Zongcheng Miao, Yaqin Chu, Lei Wang, Wenqing Zhu, Dong Wang

**Affiliations:** 1 School of Chemical and Environmental Engineering, Anhui Polytechnic University, Wuhu 241000, China; miaozongcheng@ahpu.edu.cn (Z.M.); yaqinjucinda@163.com (Y.C.); zwq_1021@163.com (W.Z.); 2 Key Laboratory of Auxiliary Chemistry & Technology for Chemical Industry, Ministry of Education, Shaanxi University of Science & Technology, Xi’an 710021, China; 3 School of Materials Science and Engineering, University of Science and Technology Beijing, Beijing 100083, China

**Keywords:** energy level, click chemistry, nonlinear optical, sensing detection

## Abstract

The molecular structure, luminescence behavior, and electronic energy level of an organic optoelectronic materials are important parameters for its synthesis. The electro-optical properties can be changed by modifying the structure of the molecule to make the electronic energy level adjustable. In this article, a series of organic conjugated micro-molecules are successfully synthesized by linking small compound units. This metal-free [2 + 2] click chemistry process generates donor–acceptor chromophore substances with high yield, high solubility, and adjustable energy levels, which can be widely used for sensors and nonlinear optics in different fields. A-TCNE, A-TCNQ, and A-F4-TCNQ molecules are characterized comprehensively via UV-Vis-NIR spectra, ^1^H NMR spectra, infrared spectroscopy, and mass spectrometry. The unique nonlinear optical phenomena and powerful intra-molecular charge–transfer interactions of these new materials give them fascinating potential for application as optoelectronic materials.

## 1. Introduction

The precise docking of small cell groups to form highly stereoselective compounds is at the heart of click chemistry, which has been applied to the synthesis of conjugated molecules to obtain efficient and stable products [1]. This novel concept, introduced by Sharpless, is a modular linkage method that allows the target compounds to be aligned perfectly and quickly in the same manner as “keychain” reactions, while avoiding the generation of harmful by-products and sensitivity to water and oxygen [2]. Cu(I)-catalyzed azide–alkyne cycloaddition reactions (CuAAc) have received a lot of attention and have numerous applications [3,4,5,6]. The Cu(I) catalyst plays an important role in lowering the energy barrier during the whole transition metal ring formation process [7,8], with the subsequent formation of triazole derivatives by ring contraction and protonation, which has helped in the development of antibacterial and anticancer drugs and ion sensors [9,10,11,12,13].

Click chemistry is a high selectivity strategy that can be combined with ion sensing, which also requires precise identification to obtain satisfactory chemical sensors. The detection and identification of metal ions is valuable in addressing environmental pollution and understanding human environmental conditions [14,15]. Lin developed a fluorescent quenching chemical sensor based on CuAAc, which is a low-cost and efficient tool for the detection of Cu^2+^ [16], while Tane et al. obtained a highly selective metal cation sensor using carbazole derivatives, which was also achieved on the basis of CuAAc derivation [17,18].

In recent years, metal-free catalyzed thermal [2 + 2] cycloaddition reactions have emerged as the focal point of click reaction types [19,20,21]. They maintain the “click” behavior without metal catalysis; furthermore, in the synthesis of organic conjugated small molecules, they have energy level modulation properties and thermal stability that other click reaction types such as CuAAc lack, and are vastly used in the synthesis of optoelectronic materials [22]. The donor–acceptor chromophores with narrow band gaps are obtained via cyclization of common electron acceptors such as tetracyanoethylene (TCNE), 7,7,8,8-tetracyanoquinodimethane (TCNQ), and 2,3,5,6-tetrafluoro-7,7,8,8-tetracyanoquinodimethane (F4-TCNQ) with electron-rich alkynes in a “click-on” manner [23]. The regulation of the energy level of the conjugated molecule is carried out by the shift of the highest occupied molecular orbital (HOMO) and lowest occupied molecular orbital (LUMO) [24,25]. Photoelectric materials are significantly favored by thermal [2 + 2] click reactions, where the products acquire strong charge–transfer effects through the optimization of electronic states, while the active photoelectric properties are suitable for devices in multiple fields, such as photovoltaic cells, ion sensors, and nonlinear optical materials [26,27,28,29,30,31,32].

In this study, conjugated photoelectric small molecules with strong redox activity are obtained by introducing electron acceptor molecules on the side chain alkyne group of 4-((2,5-dibromophenyl) ethynyl)-N, N-dihexadecylaniline with no by-products, while the quantitative consumption of the side chain alkyne group proved the high efficiency and selectivity of the click reaction. The nonlinear optical properties, electrochemical properties, and relative sensing ability of the products are described, showing the potential of the “click” method for application in optoelectronic functional materials.

## 2. Materials and Methods

### 2.1. Materials

The chemicals required for the synthesis were purchased from J&K (Beijing, China) and Aldrich (Shanghai, China). The 2,5-dibromoaniline was subjected to diazotization reaction and Sandmeyer reaction (a) to produce 2,5-dibromoiodobenzene, which was then metal coupled with 4-ethynyl-N, N-dihexadecylaniline (b) to obtain the electron-rich 4-((2,5-dibromophenyl) ethynyl)-N, N-dihexadecylaniline, with the specific synthesis procedure based on the literature method [28,33,34].

^1^H NMR spectra were measured with samples held at 20 °C on a AV300 NMR spectrometer (300 MHz, Bruker, Karlsruhe, German) while describing the resonance multiplicity of the product, with SiMe_4_ as the solvent. Infrared spectra (IR) were acquired using a JASCO FT/IR-4100 spectrometer. MALDI-TOF-MS spectra were recorded on a AXIMA-CFR mass spectrometer manufactured by Shimadzu, Kyoto, Japanat 20 kV accelerating potential with dithranol base in linear positive ion mode. UV-Vis-NIR spectra were obtained on a Shimadzu JASCO V-570 spectrophotometer. The elemental analysis was performed using a Flash EA 1112 instrument (Thermo, Massachusetts, U.S.). Electrochemical studies were taken using Ag/Ag^+^/CH_3_CN/Bu_4_NPF_6_ as the reference electrode for cyclic voltammetry measurements. The iron–ferrocene ($F_{c}/F_{c}^{+}$) couple as the potential standard was also used to obtain specific data on a CHI 660C instrument (Shanghai Chenhua Instruments Co., Shanghai, China) at rt. The Z-scan curves depict the nonlinear optical properties (NLO) and the required laser pulses were powered by a mode-locked Nd:YAG laser (EKSPLA: PL2143B).

### 2.2. Methods

TCNE (25.6 mg, 0.2 mmol) was added in a solution of 1,2-dichloroethane (5 mL) dissolved in A (79.7 mg, 0.1 mmol) to a round-bottom flask and then the mixture was stirred at 25 °C for 1 h. The solvent was removed in a vacuum and the crude product was purified via column chromatography of SiO_2_ and CH_2_Cl_2_ to give A-TCNE (87.9 mg, 95%) as a brown solid. ^1^H NMR (CDCl_3_, 300 MHz): δ = 0.90 (m, 6H), 1.29 (s, 52H), 1.58 (m, 4H), 3.39 (m, 4H), 6.68 (d, *J* = 5.7 Hz, 2H), 7.57 (d, *J* = 5.1 Hz, 1H), 7.60 (s, 1H), 7.63 (d, *J* = 5.1 Hz, 1H), 7.68 (d, *J* = 5.4 Hz, 2H) ppm. FT-IR (KBr): ν = 2922, 2853, 2216, 1602, 1487, 1416, 1367, 1340, 1207, 1183, 1030, 821, 722 cm^−1^. MALDI-TOF-MS (dithranol) *m*/*z*: calcd for C_52_H_73_Br_2_N_5_: 925.42 g·mol^−1^, found: 926.3 g·mol^−1^ [MH] ${\left[\mathrm{MH}\right]}^{+}$.Elemental analysis calcd (%) for: C 67.30, H 7.93, N 7.55; found: C 67.27, H 7.96, N 7.57.

TCNQ (40.8 mg, 0.2 mmol) was added to A (79.7 mg, 0.1 mmol) in 1,2-dichloroethane (5 mL), and the mixture was stirred at 60 °C for 1 h. The solvent was removed in a vacuum and the crude product was purified by column chromatography (SiO_2_, CH_2_Cl_2_) to obtain a greenish-black A-TCNQ (97.1 mg, 97%) solid. ^1^H NMR (CDCl_3_, 300 MHz): δ = 0.90 (m, 6H), 1.36 (s, 52H), 1.64 (m, 4H), 3.39 (m, 4H), 6.66 (d, *J* = 5.4 Hz, 2H), 7.22 (d, *J* = 5.4 Hz, 4H), 7.56 (s, 1H), 7.60 (d, *J* = 5.1 Hz, 1H), 7.63 (d, *J* = 5.1 Hz, 1H), 7.68 (d, *J* = 5.4 Hz, 2H) ppm. FT-IR (KBr): ν = 2923, 2852, 2204, 1604, 1582, 1521, 1457, 1399, 1367, 1364, 1324, 1182, 1095, 884, 827, 722 cm^−1^. MALDI-TOF-MS (dithranol) *m*/*z*: calcd for C_58_H_77_Br_2_N_5_: 1001.45 g·mol^−1^, found: 1002.3 g·mol^−1^ ${\left[\mathrm{MH}\right]}^{+}$. Elemental analysis calcd (%) for: C 69.38, H 7.73, N 6.97; found: C 69.34, H 7.77, N 6.96.

F4-TCNQ (55.2 mg, 0.2 mmol) was added at 80 °C to a solution of A (79.7 mg, 0.1 mmol) in 1,2-dichloroethane (5 mL) by stirring the mixture for 1 h. After removal of the solvent in vacuum, a purified crude product was made using column chromatography (SiO_2_, CH_2_Cl_2_) to obtain A-F4-TCNQ (97.7 mg. 91%) as a scarlet red solid. ^1^H NMR (CDCl_3_, 300 MHz): δ = 0.91 (m, 6H), 1.32 (s, 52H), 1.58 (m, 4H), 3.42 (m, 4H), 6.66 (d, *J* = 5.4 Hz, 2H), 7.55 (d, *J* = 4.8 Hz, 1H), 7.62 (s, 1H), 7.67 (d, *J* = 5.4 Hz, 1H), 7.72 (d, *J* = 5.4 Hz, 2H) ppm. FT-IR (KBr): ν = 2924, 2853, 2197, 1632, 1598, 1433, 1388, 1355, 1192, 1044, 973, 818, 720 cm^−1^. MALDI-TOF-MS (dithranol) *m*/*z*: calcd for C_58_H_73_Br_2_F_4_N_5_: 1073.42 g·mol^−1^, found: 1074.7 g·mol^−1^ ${\left[\mathrm{MH}\right]}^{+}$. Elemental analysis calcd (%) for: C 64.74, H 6.s84, N 6.51; found: C 64.70, H 6.86, N 6.54.

## 3. Results

**Figure 1:**
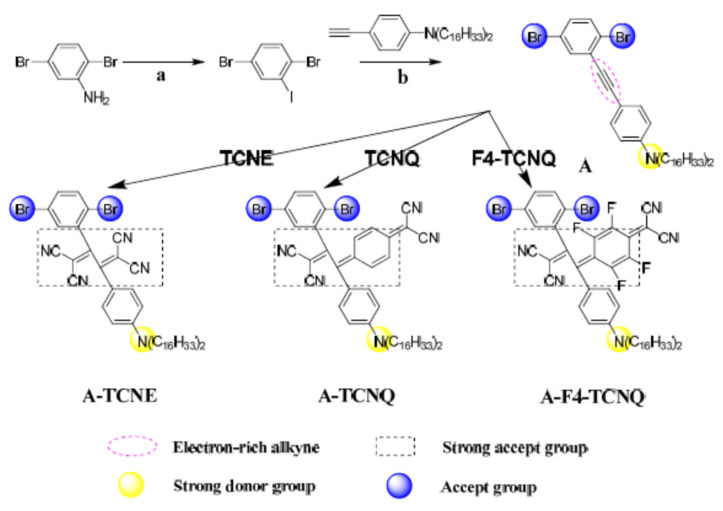
Synthesis routes of click compounds: (**a**) NaNO_2_, KI, HCl/H_2_O; (**b**) Pd(PPh_3_)_4_, CuI, Et_3_N/THF, rt.

A series of unique nonplanar donor–acceptor push–pull chromophores were synthesized from A with olefins such as TCNE, TCNQ, and F4-TCNQ attached with strongly electron-absorbing groups through click reactions. Compound A is one of the ingredients used in the click chemistry synthesis process, which is obtained from 2,5-dibromoaniline via the substitution of the amino position to an equivalent amount of the alkyne group after the Sandmeyer reaction and the catalytic coupling with Cu(Ι) (Figure 1). As shown in Figure 1, the click reagents were reacted with the pink mark acetylenic bonds, after which the samples turned dark. The synthesis of donor–acceptor chromophores was performed using aromatic precursors (strong donor groups) substituted by amines, and the reaction rates for the different click reagents strongly depended on the concentration and temperature. Practically, it was proven that high-yielding products can be produced using accurate click-type synthesis methods without generating by-products. The NMR and IR spectra and mass spectrometry data for the products also provided evidence of their good purity. Together with their promising solubility in common solvents such as acetone, these results greatly facilitate the post-processing and further use of click products to a considerable extent for modular synthesis. Distinct from conventional click chemistry, the present work does not cover the participation of metals beyond fulfilling the basic click reaction characteristics, making this a new promising type of click chemistry. This type of reaction promises as much electron uptake as possible in a narrow potential range, which is of great importance for the further optimization of optoelectronic materials and their properties.

## 4. Discussion

### 4.1. Spectral Analysis

**Figure 2:**
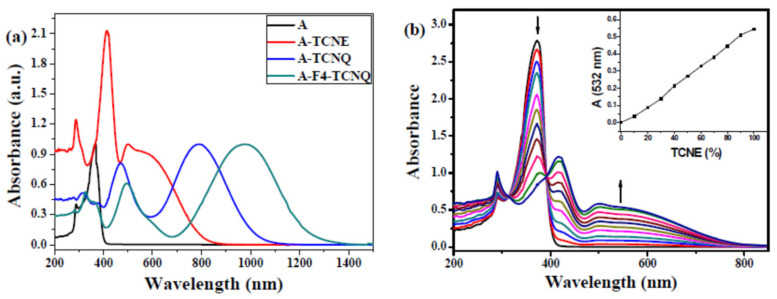
(**a**) Normalized UV-Vis-NIR spectra of compounds in CH_2_Cl_2_. (**b**) UV-Vis-NIR spectral change of A with the addition of TCNE reaction to the electron-rich alkyne. Inset: Plot of TCNE addition vs. absorbance increase at 532 nm.

The UV-Vis-NIR absorption spectra of the click derivatives prepared from A and click reagents measured in CH_2_Cl_2_ solution are shown in Figure 2a. Compared to A, A-TCNE, A-TCNQ, and A-F4-TCNQ show a clear broadening of the absorption band as well as a strong CT band with end-absorption wavelengths up to the NIR region. The strongly electron-absorbing group present in TCNE is attributed to the red shift of the plot of the A-TCNE product relative to A. In addition, the red shift of A-TCNQ is caused by the lengthening of the conjugation length of the TCNQ molecular backbone. The fluorine atom introduced in F4-TCNQ further enhanced the electron absorption ability of the group, which resulted in a remarkable strengthening of its electron affordability, with the maximum absorption peak located at 979 nm and the terminal absorption reaching 1433 nm. In conclusion, the introduction of different click molecules in A shifted the CT band from 366 to 506, 790, and 979 nm, while the terminal absorption wavelengths shifted from 434 to 925, 1180, and 1433 nm, respectively.

A quantitative TCNE solution dissolved in 1,2-dichloroethane was added dropwise at room temperature to A dissolved in CH_2_Cl_2_, which had a yellow color. The liquid then shifted to a dark red color followed by a gradual increase at about the 532 nm CT band. The isotopic points corresponding to 312 nm and 392 nm in the UV-Vis-NIR spectral region are strong evidence for the absence of side reactions, indicating that a complete reaction was carried out, consistent with click chemistry (Figure 2b). An equivocal reaction between the alkyne group of compound A and TCNE occurred, as shown in the inset in Figure 2b, since the addition of TCNE and the absorbance of A at 532 nm in the figure exhibit a linear variation.

### 4.2. Electrochemical Properties

**Figure 3:**
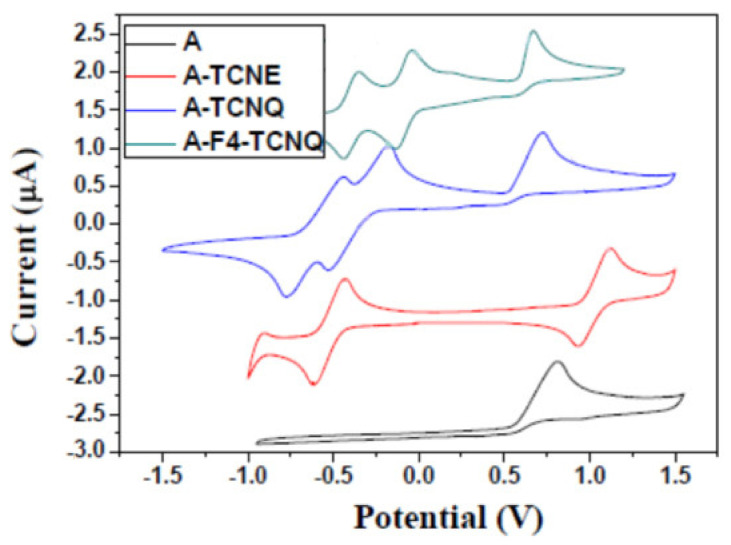
Cyclic voltammograms of all compounds in CH_2_Cl_2_ with 0.1 M Bu_4_NPF_6_ at rt, at a scanning rate of 0.1 V·S^−1^, with Ag/AgCl as the reference electrode.

To express the influence of different extents of the π-conjugated system on the redox activity of the donor–acceptor chromophore, the electrochemical properties of the A and click derivative products were characterized using cyclic voltammetry and density flooding theory (DFT). The cyclic voltammograms of the A and target products are shown in Figure 3. Table 1 provides their oxidation and reduction potentials together with the HOMO and LUMO orbital energy levels and electrochemical band gaps (Eg). The energy levels of A, A-TCNE, A-TCNQ, and A-F4-TCNQ were calculated from −4.8 eV of ferrocene (*F_c_*) with respect to the vacuum (0 eV). The oxidation potential of *F_c_* (the reference electrode was Ag/AgCl) was tested at 0.21 V. The energy level calculation formulas were:

(1) $$E_{HOMO}=-e\left[U_{on}{}^{ox}-U_{1/2,F_{c}}+4.8\;V\right]$$

(2) $$E_{LUMO}=-e\left[U_{on}{}^{red}-U_{1/2,F_{c}}+4.8\;V\right]$$

where $U_{1/2,F_{c}}$ is standardized to the semi-wave potential of $F_{c}/F_{c}^{+}$ [35,36].

From Table 1, it can be seen that the onset reduction potentials (E_on_^red^) of A-TCNE, A-TCNQ, and A-F4-TCNQ gradually increase and the LUMO orbital energy level gradually decreases. The E_on_^red^ values of A-TCNQ and A-F4-TCNQ increase by 0.15 eV and 0.33 eV, whereas the LUMO orbital energy levels decrease by 0.15 eV and 0.33 eV over that of A-TCNE. This illustrates that the electron-accepting ability of the click derivatives is affected by the stretch of the π-conjugation structure and the addition of strong electron-absorbing groups, which makes it easier to form the donor chromophore fraction. Both A-TCNQ and A-F4-TCNQ exhibit lower Eg values than the A-TCNE, possibly because of the π-conjugation depletion of the acceptor molecules. In most cases, these electrochemical band gaps are well in accordance with the optical band gaps determined by terminal absorption.

### 4.3. Nonlinear Optical Properties

**Figure 4:**
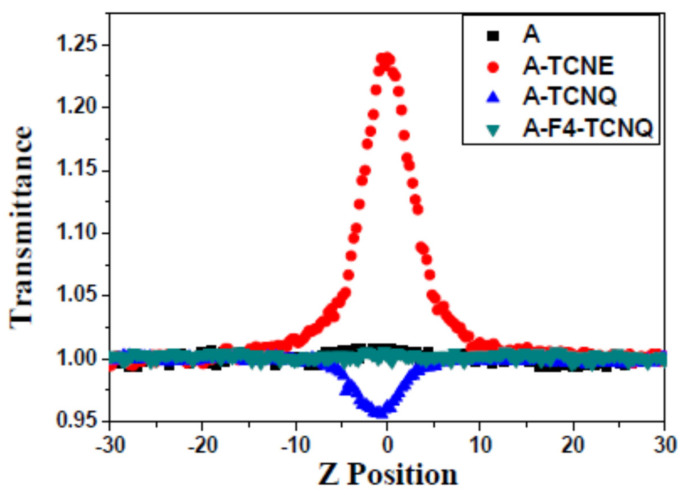
Z-scan results of the compounds in CH_2_Cl_2_.

The Z-scan technique is an important method relying on the spatial distortion of the beam to study the nonlinear optical properties (NLO) of the target product. Figure 4 shows the NLO results of the click products produced by the reaction of different click reagents without nonlinear optical phenomena. Additional respective parameters are shown in Table 1. After being clicked by TCNE, A-TCNE obviously exhibited nonlinear saturable absorption because of the strong absorption intensity of A-TCNE at 532 nm. Strong proof that A-TCNE can be used as a nonlinear optical material can be found from n2<0. The values are of a similar order of magnitude to the common acridone, chalcone, and quinazolinone derivatives [37,38,39]. From A-TCNE to A-TCNQ, the nonlinear absorption coefficients β of materials were found to change from negative to larger positive values, and a trend from nonlinear saturable absorption to nonlinear inverse saturable absorption was found, which was attributed to the extended conjugation length of the molecular backbone. If F4-TCNQ was used as the click reagent, the nonlinear optical phenomena disappeared. Perhaps, as an electron-withdrawing group, the moiety from F4-TCNQ was so strong that the nonlinear optical response time was clearly shortened.

### 4.4. Sensing Detection

**Figure 5:**
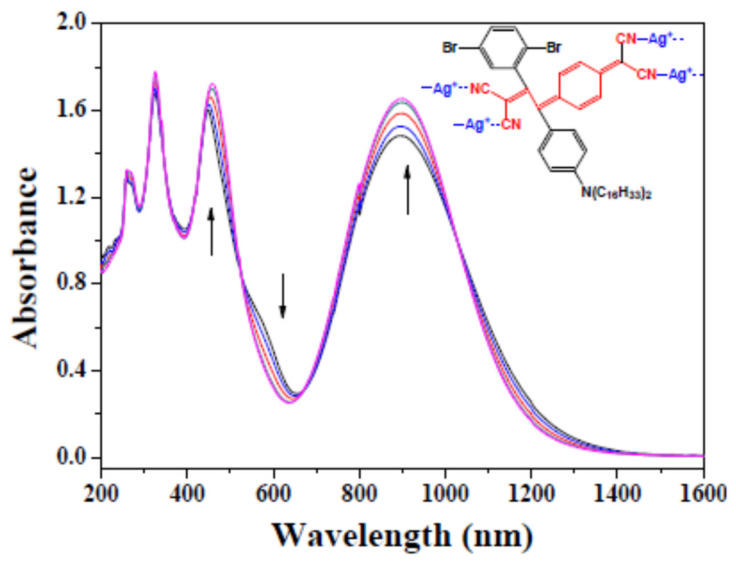
UV-Vis–NIR spectral changes of the sensors with Ag^+^ (A-F4-TCNQ/DMF: 1 × 10^−4^ mol/L; AgSO_3_CF_3_/DMF: 0.2 mol/L, titration dose: 2 μL each, rt). Inset: Intermolecular interaction between A-F4-TCNQ and Ag^+^.

The donor–acceptor sensor molecule, which is obtained via click reaction using the alkyne group as a donor and then selecting a suitable acceptor substance, has promising research potential and has a strong visible absorption band that is explained by the intra-molecular CT interactions. According to Figure 5 (Inset), when Ag^+^ was chosen as a tight receptor molecule, the inverse anions of the examined metal cations of trifluoromethanesulfonic acid (OTf) were inert in the recognition event. In DMF, the CT band had a maximum peak position at 896 nm, which was lower than in CH_2_Cl_2_. The position of the peak did not change; however, a linear increase in intensity was apparent with the amount of Ag^+^ added. A red shift of the other peak (at 450 nm) occurred as a result of an increase in the addition of Ag^+^.

On titration, the isotopic points appeared at 525 and 1027 nm, meaning that no side reactions occurred in the whole reaction system and only click reactions were present. Same titration experiments were made for other click compounds, without being able to detect particular phenomena. According to the inset in Figure 5, an individual A-F4-TCNQ molecule contains four −CN, which can theoretically react with four molecules of AgOTf in coordination [40,41], while the actual titration process also confirmed the quantitative relationship between A-F4-TCNQ and the added Ag^+^ close to 1:4, at the same time proving the interaction between −CN and Ag^+^. The phenomenon in Figure 5 also excluded the potential for intermolecular aggregation. It was also suggested that the cyanide of A-F4-TCNQ has a high electron density distribution, and may show strong electronegativity or even an ionic nature.

## 5. Conclusions

A set of substituted alkyne-acceptor molecules were synthesized in high yields via metal-free [2 + 2] cycloaddition click reactions, whereby the strength of the conjugation system in the acceptor module had a great influence on the properties of the whole molecule. The synthesized A-TCNE, A-TCNQ, and A-F4-TCNQ were characterized using ^1^H NMR, UV-Vis-NIR, and MS. When conjugated with increasing intensity of the acceptor molecules and the electron-absorbing groups, an apparent red shift in the UV-VIS-NIR spectra of the click derivatives was exhibited, which was caused by the strong intramolecular charge–transfer effect. By testing the electro-chemical properties, it was found that in combination the expansion of the π-conjugated system and the electronic absorbing groups enhanced the formation of donor chromophores in the conjugated molecules. However, the nonlinear optical effect of the material disappears in A-F4-TCNQ, which may be due to the sharp shortening of the response time of the nonlinear optics caused by multiple strong electron-absorbing groups (–CN), thereby exhibiting a nonlinear optical phenomenon different from that of A-TCNE and A = TCNQ. In particular, there are direct recognition sites for Ag^+^ in the donor–acceptor chromophores. In conclusion, the new materials synthesized in this paper are of high application value as optoelectronic materials in terms of their modulation performance, sensing detection, and nonlinear optics.

## Figures and Tables

**Table 1:** Optical and electrochemical properties of the compounds.

Materials	λ (nm)	E_on_^ox 1^ (V)	E_on_^red 1^ (V)	HOMO (eV)	LOMO (eV)	Eg ^2^ (eV)	Eg ^3^ (eV)	β (×10^−11^ m/W)	n_2_ (×10^−18^ m^2^/W)
A	366	0.54	-	−5.13	-	-	2.86	-	-
A-TCNE	414,506	0.96	−0.45	−5.55	−4.14	1.41	1.34	−3.6	−2.4
A-TCNQ	470,790	0.52	−0.30	−5.11	−4.29	0.82	1.05	43.0	-
A-F4-TCNQ	494,979	0.55	−0.12	−5.14	−4.47	0.67	0.87	-	-

^1^ Onset potentials determined from cyclic voltammograms. ^2^ Band gaps calculated from the energy levels of cyclic voltammograms. ^3^ Band gaps estimated from the end-absorption wavelengths of optical absorption in CH_2_Cl_2_ solution.
